# *Candida* bloodstream infection under veno-arterial ECMO therapy

**DOI:** 10.1186/s13054-019-2593-4

**Published:** 2019-09-17

**Authors:** Quentin de Roux, Françoise Botterel, Raphaël Lepeule, Fabio Silvio Taccone, Olivier Langeron, Nicolas Mongardon

**Affiliations:** 1 Service d’anesthésie-réanimation chirurgicale, réanimation chirurgicale cardio-vasculaire, DMU CARE, Assistance Publique des Hôpitaux de Paris (APHP), Hôpitaux Universitaires Henri Mondor, 51 avenue du Maréchal de Lattre de Tassigny, 94000 Créteil, France; 2 U955-IMRB, Equipe 03 “Stratégies pharmacologiques et thérapeutiques expérimentales des insuffisances cardiaques et coronaires”, Inserm, UPEC, Ecole Nationale Vétérinaire d’Alfort, Maisons-Alfort, France; 3 Unité de Parasitologie-Mycologie, Département des agents infectieux, Assistance Publique des Hôpitaux de Paris (APHP), Centre Hospitalier Universitaire Henri Mondor, Créteil, France; 4 Université Paris Est Créteil, Faculté de Médecine, Créteil, France; 5 Unité transversale de traitement des infections, Département des agents infectieux, Assistance Publique des Hôpitaux de Paris (APHP), Centre Hospitalier Universitaire Henri Mondor, Créteil, France; 6 Department of Intensive Care, Clinique Universitaire de Bruxelles (CUB) Erasme, Université Libre de Bruxelles, Bruxelles, Belgium

Cavayas et al. recently described invasive fungal infections in patients under extra-corporeal membrane oxygenation (ECMO) of the Extracorporeal Life Support Organization registry [1]. They found a 1.2% prevalence of *Candida* bloodstream infections (BSI). However, we highlighted the heterogeneity of this mixed population treated with veno-venous (VV) and veno-arterial (VA) ECMO and the scarce available data, in particular the delay from ECMO to infection [2].

As no specific report is available on *Candida* BSI under VA-ECMO, we investigated the incidence and timing of this complication in our large database of 150 VA-ECMO (January 2013 to January 2017) who survived more than 24 h (Table 1). Our surveillance protocol includes systematic blood culture (BC) once daily, since ECMO implantation up to 5 days after support withdrawal. Of the 2163 BC samples collected, either as routine or “on-demand” by the attending physician, 192 were positive; after exclusion of contaminants, 117 BC (61%) were related to bacterial infection in 46 patients. Only 7 (0.04%) were positive for yeasts, for a total of 5 BSI episodes in 4 patients. BSI rate was 43 cases/1.000 days of ECMO support. All yeast BSI were positive for *Candida* spp. In all cases, candidemia occurred in the third week after VA-ECMO implantation: delay between ECMO implantation and first positive BC was 17 [16–19] days. In one patient, candidemia occurred 4 days after ECMO withdrawal. Weaning from ECMO occurred in all 4 patients after 20 [17–21] days; moreover, only the patient with candidemia after ECMO withdrawal survived. Among the 46 patients with bacterial BSI, 27 died.

**Table 1:** Patients characteristics

	All patients (*n* = 150)	Patients with *Candida* BSI (*n* = 4)
Age (years)	58 [48–69]	50 [40–57]
BMI (kg/m^2^)	25 [23–29]	28 [26–29]
Long-term corticosteroid therapy	7 (5)	0 (0)
SAPS II	54 [38–70]	41 [35–49]
SOFA score at day 0	13 [11–15]	12 [12–12]
Arterial lactate level at day 0 (mmol/L)	5 [3–9]	6 [3–10]
VA-ECMO for post-cardiotomy shock	65 (43)	2 (50)
VA-ECMO for medical reason	85 (57)	2 (50)
Refractory cardiac arrest	39 (26)	1 (25)
Graft failure after heart transplantation	14 (9)	0 (0)
Intra-aortic balloon pump	26 (17)	0 (0)
KDIGO stage at day 0		
0	52 (35)	2 (50)
1	40 (27)	0 (0)
2	21 (14)	1 (25)
3	32 (22)	1 (25)
RRT during VA-ECMO course	63 (42)	3 (75)
Red blood cell units at day 1	4 [0–8]	9 [5–12]
Upper gastrointestinal tract bleeding	22 (14)	3 (75)
Enteral nutrition in the first 5 days	128 (86)	4 (100)
Acute mesenteric ischemia during ECMO course	14 (9)	2 (50)
Antimicrobial therapy during ECMO course	134 (89)	4 (100)
Pneumonia during VA-ECMO support	76 (51)	0 (0)
At least one extra-pulmonary infection during VA-ECMO support	68 (45)	3 (75)
VA-ECMO support duration (days)	7 [5–13]	20 [17–21]
Mechanical ventilation duration (days)	14 [6–27]	19 [18–22]
ICU mortality	84 (56)	3 (75)

Data are expressed as median [interquartile 25–75] or number (percentage), as appropriate

Abbreviations: *BMI* body mass index, *BSI* bloodstream infection, *VA-ECMO* veno-arterial extra-corporeal membrane oxygenation, *SAPS II* Simplified Acute Physiology Score II, *SOFA* Sequential Organ Failure Assessment, *KDIGO* Kidney Disease: Improving Global Outcomes, *RRT* renal replacement therapy, *ICU* intensive care unit

Prevalence of invasive *Candida* disease ranges from 0 to a third of patients under ECMO [3, 4]. However, studies included both VV and VA-ECMO, without clear distinction between *Candida* BSI and other forms of *Candida* infections and without data on the timing of BSI occurrence. Moreover, whether BC were performed systematically or on-demand remains unknown. While it was difficult to draw conclusions with these heterogeneous populations, our results highlight that *Candida* BSI is rare and occurs late during the ECMO course. On the opposite, early septic shock under VA-ECMO is frequent and largely due to bacteria [5]. Consequently, antifungal therapy should not be part of the first-line empiric antimicrobial therapy in case of septic shock occurring within the first 2 weeks of ECMO support, unless indicated for other conditions (i.e., prolonged febrile neutropenia or tertiary peritonitis). Sepsis under prolonged mechanical support should raise the possibility of invasive *Candida* infection.

## Data Availability

Available on request at nicolas.mongardon@aphp.fr
